# Dynamic Coordination of Alternative Splicing and Subgenome Expression Bias Underlies Rusty Root Symptom Response in *Panax ginseng*

**DOI:** 10.3390/plants14142120

**Published:** 2025-07-09

**Authors:** Jing Zhao, Juzuo Li, Xiujuan Lei, Peng Di, Hongwei Xun, Zhibin Zhang, Jian Zhang, Xiangru Meng, Yingping Wang

**Affiliations:** 1 College of Chinese Medicinal Materials, Jilin Agricultural University, Changchun 130118, China; zhaoj878@nenu.edu.cn (J.Z.); xiujuanl@jlau.edu.cn (X.L.); di@jlau.edu.cn (P.D.); 2 State Local Joint Engineering Research Centre of Ginseng Breeding and Application, Jilin Agricultural University, Changchun 130118, China; 3 Department of Biology, School of Life Sciences, Southern University of Science and Technology, Shenzhen 518055, China; lijz3@sustech.edu.cn; 4 Key Laboratory of Molecular Epigenetics of the Ministry of Education (MOE), Northeast Normal University, Changchun 130024, China; xunhw334@nenu.edu.cn (H.X.); zhangzb554@nenu.edu.cn (Z.Z.); 5 Faculty of Agronomy, Jilin Agricultural University, Changchun 130118, China; 6 Department of Biology, University of British Columbia, Okanagan, Kelowna, BC V1V 1V7, Canada

**Keywords:** *Panax ginseng*, ginseng rusty root symptoms, post-transcriptional regulation, alternative splicing, subgenome expression bias

## Abstract

Ginseng rusty root symptoms (GRSs) compromise the yield and quality of *Panax ginseng*. While transcriptomic analyses have demonstrated extensive remodeling of stress signaling networks, the post-transcriptional defense circuitry remains obscure. We profiled alternative splicing (AS) in three phloem tissues, the healthy phloem (AG), the non-reddened phloem neighboring lesions (BG), and the reddened lesion core (CG), to delineate AS reprogramming during GRS progression. The frequency of AS was sharply elevated in CG, with intron retention predominating. Extensive gains and losses of splice events indicate large-scale rewiring of the splice network. Overlapping differentially alternative spliced genes (DAGs) identified in both CG vs AG and CG vs BG contrasts were significantly enriched for RNA–spliceosome assembly and stress–response pathways, revealing a conserved post-transcriptional response associated with lesion formation. Integrative analysis of differentially expressed genes uncovered 671 loci under dual regulation; functional classification categorized these genes in receptor-like kinase signaling and chromatin-remodeling modules, underscoring the synergy between AS and transcriptional control. Moreover, the B subgenome disproportionately contributed stress-responsive transcripts in diseased tissue, suggesting an adaptive, subgenome-biased strategy. These findings demonstrate that dynamic AS remodeling and subgenome expression bias jointly orchestrate ginseng defense against GRS and provide a framework for breeding disease-resilient crops.

## 1. Introduction

*Panax ginseng* C.A. Meyer, revered as the “King of Herbs”, is a shade-adapted medicinal plant whose bioactive constituents, notably ginsenosides and polysaccharides, deliver neuroprotective, antineoplastic, and immunomodulatory benefits [1,2,3]. Existing research indicates that all angiosperms have undergone one or more polyploidization events [4]. Notably, *P. ginseng* has experienced two polyploidization events during its evolution, classifying it as an allotetraploid plant [5,6]. Its complex genome comprises 69,242 genes, encompassing 4788 Pfam domains, which are distributed across two distinct subgenomes, the A subgenome and B subgenome [5]. A significant difference exists in the proportion of genes associated with these Pfam domains between the two subgenomes relative to their total gene counts, with the A subgenome accounting for 3.15% and the B subgenome for 2.90% [5]. This highlights the divergent gene functions between the subgenomes, underscoring the genetic complexity that may underpin both its unique medicinal properties and its specific environmental vulnerabilities [5]. However, commercial cultivation is hampered by the species’ protracted growth cycle and narrow edaphic niche: ginseng thrives only in slightly acidic, moist, well-drained soils and is therefore exceptionally prone to soil-borne diseases that depress both yield and quality [7,8].

Ginseng rusty root symptoms (GRSs) are a destructive root disorder encountered in field cultivation [9,10]. If the soil has too much moisture and organic matter does not rot quickly enough, this can cause GRS [11]. The disease can produce rust-colored lesions on the root epidermis initially and, under severe infection, progress to cortical decay and plant death [12]. Pathological studies indicate that diseased tissues are enriched with iron–aluminum complexes (e.g., iron oxides, aluminosilicates, and organic aluminum complexes). The Fe^3+^ and Al^3+^ ions released from these complexes are closely related to the formation of symptoms [7,13]. Regarding the pathogenic mechanism of ginseng rusty root (GRS), the current understanding points to two main causes [14,15]. First, GRS is often viewed as a physiological disorder, an abiotic stress response provoked by unfavorable edaphic factors such as aluminum toxicity, heavy-metal accumulation, prolonged soil moisture, or nitric oxide stress [11,12,16,17]. Second, GRS can also be attributed to defensive responses to intruding microorganisms. For instance, the formation of a rusty color can be induced by bacteria, including *Microbacterium oxydans* and *Rhizobium leguminosarum*, and pectinase with iron (Fe), synergistically enhancing the severity of the rust symptoms [18,19]. Moreover, the invasion of weakly aggressive *Ilyonectria* species has been postulated as a cause of rusty root, with their growth potentially stimulated by the available iron in ginseng [13,14,20]. Furthermore, abiotic triggers such as sustained high humidity, heavy-metal accumulation, and shifts in the rhizosphere microbiome aggravate GRS by disrupting root physiology [11,21,22,23]. Transcriptome surveys have shown that GRS activates canonical defense pathways, including phenylpropanoid metabolism and hormone signaling, yet the molecular circuitry that fine-tunes these responses, particularly at the post-transcriptional level, remains obscure [9,21,24].

Alternative splicing (AS) has emerged as an important layer of immune regulation in plants, generating functionally divergent isoforms through exon skipping (ES), alternative donor (A5), alternative acceptor (A3), and intron retention (IR) [25,26,27]. Previous studies have elucidated its dual role in host–pathogen interactions: in wheat, exon skipping of the *Pm4* gene produces a chimeric kinase isoform conferring race-specific resistance to powdery mildew [28]; the *Phytophthora infestans* effector Avrblb1 hijacks the splicing factor StCWC15 to suppress intron retention of the potato *RB* gene, thereby promoting full-length resistance protein accumulation [29]; and in cotton, the splicing factor *SR45a* enhances *Verticillium dahliae* resistance by modulating AS of immunity-related *PR1* [30]. Notably, AS also critically mediates plant adaptation to abiotic stresses: in *Arabidopsis*, heat stress-induced alternative polyadenylation of the DREB2A transcript generates a truncated isoform that fine-tunes drought-responsive gene expression to balance growth and stress tolerance [31]. These findings underscore the versatility of AS in not only enhancing plant immunity but also being subverted by pathogens, while serving as a molecular nexus at the intersection of biotic and abiotic stress cross-talk. Nevertheless, whether AS participates in the defense against ginseng rusty root syndrome (GRS) remains unexplored.

In *P. ginseng*, leveraged PacBio single-molecule Iso-Seq on root, stem, leaf, and flower tissues to generate 135,317 high-quality full-length transcripts (mean length ≈ 3.2 kb) uncovered that nearly half of the isoforms were previously unannotated, and markedly improved isoform completeness and coding-sequence accuracy over short-read assemblies [32]. However, these long-read data were obtained under non-stress conditions and did not interrogate defense-related AS, leaving the contribution of splice variation to GRS resistance entirely unresolved.

Here, we characterize the genome-wide landscape of AS reprogramming in ginseng roots infected by GRS, including healthy (AG), lesion-adjacent (BG), and lesion-core (CG) periderm tissues. By integrating alternative splicing, gene expression regulation, and subgenome bias, we aim to clarify the multidimensional regulatory network of AS in GRS pathogenesis. This research will provide a novel insight for understanding the molecular mechanisms of disease resistance in ginseng and offer novel insights for splice-targeted crop improvement strategies.

## 2. Results

### 2.1. Global Changes in Ginseng Root Alternative Splicing Patterns Induced by Rusty Root Symptom Infection

Alternative splicing (AS) is known to regulate plant RNA silencing and immune responses [17,18,19]. To investigate the impact of GRS infection on AS patterns in *P. ginseng* roots, we conducted a comparative analysis of genome-wide AS patterns using transcriptome data from healthy *P. ginseng* phloem tissue (AG), the non-red tissue of the phloem epidermis around the lesion (BG), and the red lesion site tissue of GRS (CG). Our analysis identified 19,723, 19,279, and 20,279 alternative splicing events (ASEs) in AG, BG, and CG, respectively. These ASEs corresponded to 13,202, 12,936, and 14,059 alternative spliced genes (ASGs) in AG, BG, and CG, respectively (Figure S1 and Tables S1 and S2). These results indicate that the CG samples exhibited a higher number of both genome-wide AS events and associated genes compared to AG and BG, suggesting that GRS infection induces the occurrence of AS in severely infected ginseng root tissues.

**Figure 1:**
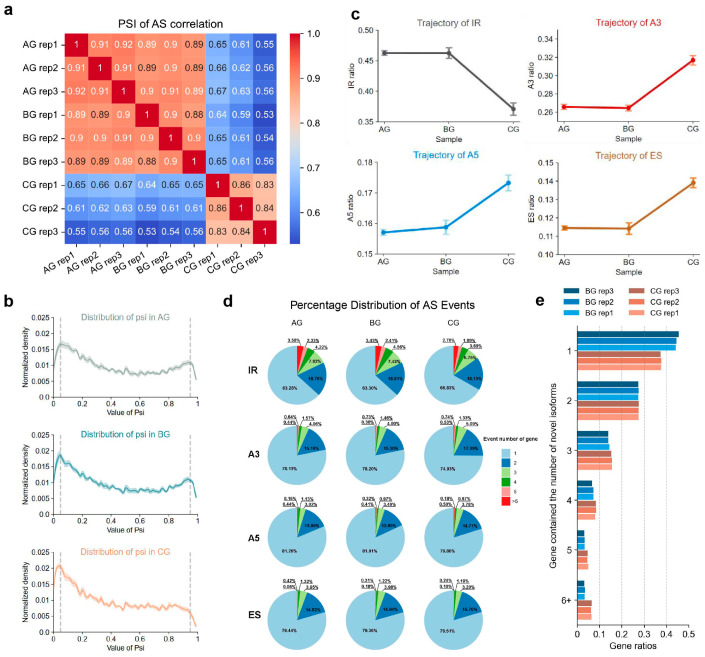
Characteristics of alternative splicing in healthy *Panax ginseng* phloem tissue (AG), the non-red tissue of the phloem epidermis around the lesion (BG), and the red lesion site tissue of GRS (CG). (**a**) Percentage Spliced In (PSI) correlation of AS events. This heatmap displays the correlation matrix of PSI for alternative splicing (AS) events across different samples (AG, BG, and CG) and their replicates. Each cell represents the correlation coefficient between two replicates or samples, with values ranging from 0 (blue) to 1 (red). (**b**) Density distribution of PSI in AG, BG, and CG. The *x*-axis represents PSI, and the *y*-axis indicates the normalized density of these values. The plot illustrates differences in splicing behavior between sample groups. The left grey dashed line represents a PSI of 0.05, and the right grey dashed line represents a PSI of 0.95. (**c**) Frequencies of four alternative splicing event types: exon skipping (ES), intron retention (IR), alternative donor (A5), and alternative acceptor (A3) in AG, BG, and CG samples. Data points represent the mean values for each replicate, and error bars indicate the standard deviation. (**d**) Number of alternative splicing events corresponding to four types of alternative spliced genes. Light blue, dark blue, light green, dark green, pink, and red correspond to 1, 2, 3, 4, 5, and >5 alternative splicing events per alternative spliced gene, respectively. (**e**) Proportion of genes with newly formed isoforms in BG (blue) and CG (red) compared to AG. The *x*-axis shows the gene proportion, and the *y*-axis indicates the number of newly formed isoforms (1, 2, 3, 4, 5, or >5).

To evaluate the consistency of splicing patterns within and between biological replicates of AG, BG, and CG, we quantified AS using Percentage Spliced In (PSI) and performed clustering analysis [33]. The results showed a high correlation (*r* > 0.8, *p*-value < 0.05) among the three biological replicates within each of the AG, BG, and CG groups, indicating stable splicing patterns (Figure 1a). The correlation coefficients between AG and BG replicates were also relatively high (*r* = 0.88, *p*-value < 0.05), whereas those between CG and AG/BG replicates were slightly lower (maximum *r* = 0.67, *p*-value < 0.05). The results suggest a more significant difference in splicing patterns in CG compared to other two groups, implying that severe GRS infection impacts ginseng AS patterns (Figure 1a and Figure S2). A comparison of the PSI distributions for ASGs revealed significant differences between CG and both AG and BG (Kolmogorov–Smirnov test, CG vs. AG, *p*-value = 6.07 × 10^−16^; CG vs. BG, *p*-value = 2.37 × 10^−15^), while no significant difference was observed between AG and BG (Kolmogorov–Smirnov test, *p*-value = 0.09) (Figure 1b). These finds indicate that CG exhibits a distinct genome-wide splicing distribution pattern potentially reflecting a specific physiological or stress response characteristic.

Further categorization of the identified AS events into four main types, exon skipping (ES), intron retention (IR), alternative donor (A5), and alternative acceptor (A3), and subsequent frequency analysis revealed similar compositions of AS events across AG, BG, and CG. IR was predominant, accounting for 36.99–45.63% of all events, in agreement with patterns reported for many other plant species [34,35]. A3 (27.48–32.20%) and A5 (15.96–17.64%) followed, whereas ES, being the least frequent AS event, represented only 10.93–13.16% of events (Figure 1c and Figure S1a; Table S1).

Comparative analysis of these four AS event types revealed more consistent patterns between AG and BG, whereas CG displayed a distinct profile. In CG, the frequency of IR events was lower, whereas the frequencies of A5, A3, and ES events were higher to AG and BG (Figure 1c and Figure S1a; Table S1). Further analysis of the distribution of A3 and IR at the gene level in CG showed that the number of IR events was higher than A3 events (A3_CG_ vs. IR_CG_, 32.20% vs. 36.99%), while the number of genes exhibiting A3 events was higher than those with IR events (A3_CG_ vs. IR_CG_, 35.69% vs. 32.18%) (Figure S1b; Table S2). These observations imply that IR tends to recur within a restricted subset of genes, inflating its overall event count, whereas A3 events tend to occur in a broader range of genes, resulting in a more widespread impact at the gene level.

Statistical analysis of the number of AS events (ASEs) per alternative spliced gene (ASG) across AG, BG, and CG revealed that the frequency of a single ASE of ASG was the highest, with CG displaying a slightly higher proportion than AG and BG (CG vs. AG = 74.02% vs. 73.29%; CG vs. BG = 74.02% vs. 73.45%) (Tables S2 and S3). When examining the four AS categories individually, CG differed markedly from AG and BG: for A3, A5 and ES, CG harbored more genes with two or three ASEs, and a larger fraction with more than five A3 or A5 events (Figure 1d and Table S3). These results indicate that GRS infection remodels the splicing landscape of ginseng roots, with IR potentially being primarily involved in the splicing regulation of core genes, while ES or alternative splice sites might generate a greater number of transcripts, potentially supporting more complex transcriptomic diversity. Subsequent analysis of the relationship between the number of AS events and transcript diversity showed a significant positive correlation across all samples (*r*_AG_ = 0.69, *p*-value < 0.001; *r*_BG_ = 0.69, *p*-value < 0.001; *r*_CG_ = 0.69, *p*-value < 0.001) (Figure S3), demonstrating that an increase in AS events corresponds to the production of a greater variety of transcripts from individual genes. Further comparison of novel isoforms generated in BG and CG relative to AG revealed that, although most genes in both datasets produced only one or two new isoforms, and more than 40% of BG genes produced just one, CG contained a high proportion of genes generating three to five novel isoforms (Figure 1e and Figure S3). Taken together, these results indicate that CG undergoes a more dynamic progress of splicing, yielding greater transcript diversity that may contribute to genome regulation and stress adaptation.

### 2.2. GRS Infection Shifts Subgenome- and Chromosome-Specific Alternative Splicing Landscapes

**Figure 2:**
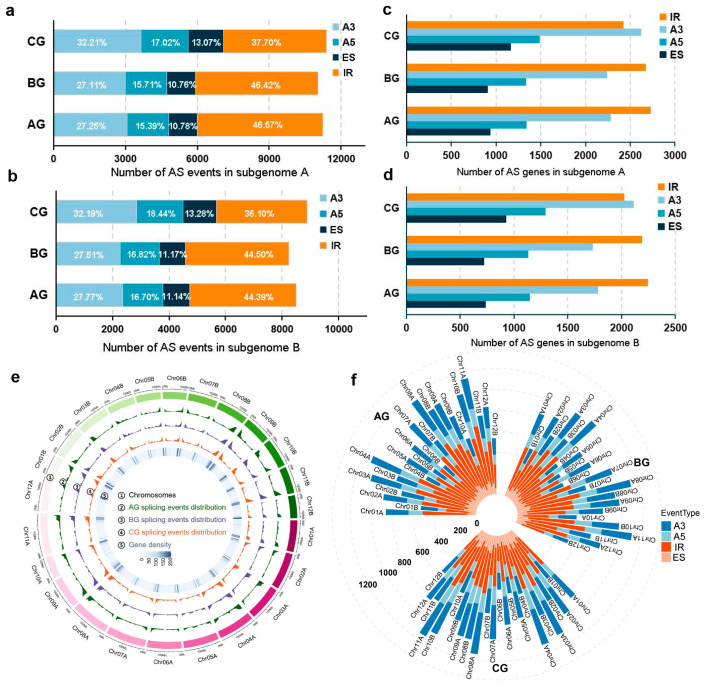
Subgenome and chromosomal level alternative splicing events in AG, BG, and CG. (**a**,**b**) Number and proportion of four types of alternative splicing events, intron retention (IR), alternative acceptor (A3), alternative donor (A5), and exon skipping (ES), in the A/B subgenomes. (**c**,**d**) Number of genes undergoing four types of alternative splicing events in the A/B subgenomes. (**e**) Distribution patterns of splicing events on 24 chromosomes. The circos plot, from outer to inner, displays the following: 1. chromosomes Chr01A–Chr12A (gradient pink) and Chr01B–Chr12B (gradient green), totaling 24 chromosomes; 2. distribution of splicing events on chromosomes in AG (green); 3. distribution of splicing events on chromosomes in BG (purple); 4. distribution of splicing events on chromosomes in CG (orange); and 5. gene density. (**f**) Chromosomal level distribution of alternative splicing (AS) events. Bar charts show the number of AS events (*y*-axis) on chromosomes of the A subgenome (chr01A–chr12A) and B subgenome (chr01B–chr12B) (*x*-axis) in AG, BG, and CG.

To dissect the differences in AS event types among AG, BG, and CG, we defined the distribution of AS event types at both the subgenome and chromosomal levels. At the subgenome level, the A subgenome consistently exhibited a higher frequency of AS events than the B subgenome across AG, BG, and CG (Figure 2a,b). In CG, the number of IRs (4295 in A subgenome; 3207 in B subgenome) and A3s (3670 in A subgenome; 2860 in B subgenome) were higher than in AG and BG (Figure 2a,b and Table S4). Concurrently, the frequency of A3 events increased in CG, while the frequency of IR events significantly decreased (Chi-square test, *p*-value < 0.01). When analyzing the number of genes undergoing AS events, we found that the number of genes associated with A3 in CG (2624 in A subgenome; 2114 in B subgenome) was significantly higher than the number of genes associated with IR (2422 in A subgenome; 2025 in B subgenome) (Chi-square test, *p*-value < 0.01) (Figure 2c,d and Table S5). These findings indicate that the changes in AS events and AS-related genes in the A and B subgenomes align with the overall genome-wide trends. Furthermore, we found that splicing events congregated within gene-rich chromosomal segments, particularly at the proximal to telomere region (Figure 2e), highlighting a tight linkage between transcriptional complexity and AS activity. At chromosomal resolution, both A3 and IR events accumulated at discrete hotspots, most notably on Chr04A, Chr08A, and Chr10B (Figure 2f). This observation may be linked to specific gene sequence characteristics or chromatin conformations in these regions, which are hypothesized to be key factors contributing to the accumulation of splicing events.

### 2.3. Differential Expression Analysis of Unique Expression Patterns and Subgenome Bias in GRS-Affected Ginseng Tissues

**Figure 3:**
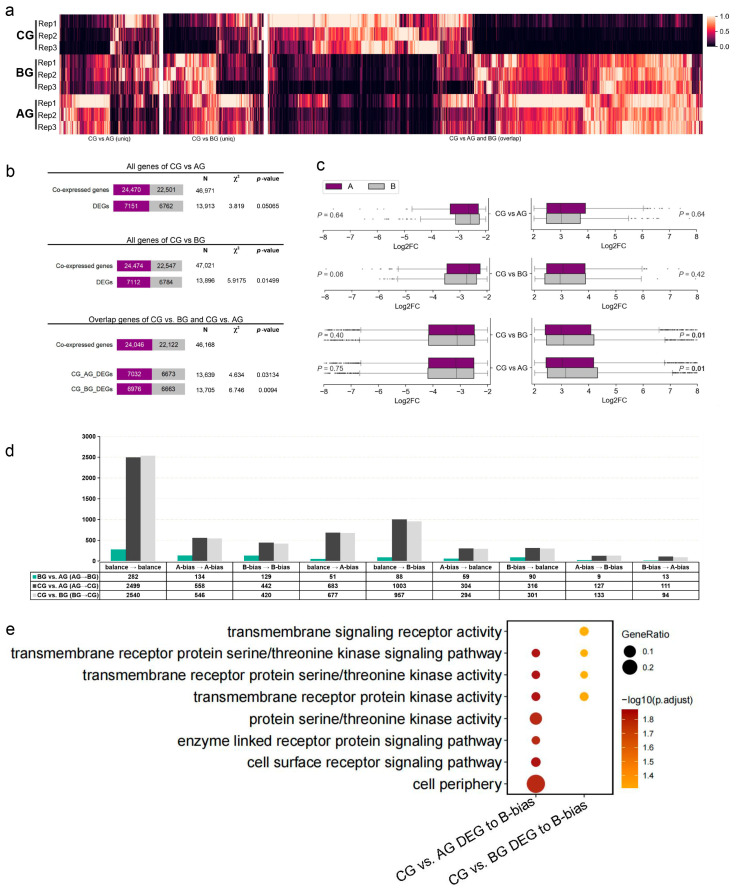
Comprehensive analysis of GRS-induced transcriptional alterations in *P. ginseng* roots. (**a**) Heatmap of uniquely expressed genes (TPM) in pairwise comparisons among samples CG, AG, and BG. The heatmap comprises three gene sets: CG vs. AG, CG vs. BG, and overlapping genes of CG vs. AG and CG vs. BG. Expression levels are represented by the colorkey (light: high TPM; dark: low TPM) with three biological replicates (Rep1–3). (**b**) Subgenome-specific DEG distribution in CG vs. AG, CG vs. BG, and their overlaps. Purple/grey bars represent A/B subgenome DEG counts (|log2FC| > 2, *p*-value < 0.05), with Chi-square values (*χ*^2^) indicating subgenomic bias significance. Numbers within the bars indicate the gene count for each subgenome; N denotes the total gene count. (**c**) Log2FC distribution of A/B subgenome DEGs. Boxplots compare expression magnitudes of downregulated (left) and upregulated (right) genes. A subgenome (purple) and B subgenome (grey). Mann–Whitney U test was used for statistical significance analysis, *p*-value < 0.05 indicates statistical significance between subgenomes. (**d**) Dynamic analysis of subgenome-biased gene expression. Transcriptome comparisons of AG, BG, and CG reveal three patterns: balanced (no subgenome dominance), A-biased, and B-biased. Numerical labels indicate the number of differentially expressed genes. (**e**) Enrichment analysis of B-biased genes in CG vs. AG and BG vs. CG comparisons. Circle size represents the GeneRatio (N/M), the number of genes from our gene list found in a specific pathway (N) to the total number of genes annotated to that same pathway in the genomic background (M); color depth indicates statistical significance (–log10 (*p*-adjust)).

To elucidate the transcriptional dynamics associated with GRS in *P. ginseng*, we performed comparative transcriptomic analyses of AG, BG, and CG. Gene expression patterns revealed robust reproducibility within each sample group. While intra-group patterns were consistent, overlapping genes between CG vs. AG and CG vs. BG comparisons displayed distinct expression profiles compared to their respective global patterns (Figure 3a). Further analysis of expression patterns revealed no significant difference in the number of differentially expressed genes (DEGs) between the two subgenomes in the CG vs. AG comparison (DEG _A subgenome_ = 7151, DEG _B subgenome_ = 6762, expressed gene _A subgenome_ = 24,470, expressed gene _B subgenome_ = 22,501, *χ*^2^ = 3.82, *p*-value = 0.051). However, a significant difference in DEG numbers was observed between the A subgenome (DEG/expressed genes = 7112/24,470) and B subgenome (DEG/expressed genes = 6784/22,501) in the CG vs. BG comparison (*χ*^2^ = 6.58, *p*-value = 0.01). Furthermore, among the overlapping DEGs between CG vs. AG and CG vs. BG, the proportion of DEGs from the B subgenome was consistently and significantly higher than that from the A subgenome (*p*-value < 0.05) (Figure 3b).

While no significant differences in the magnitude of upregulation or downregulation were found between the two subgenomes when comparing CG vs. AG and CG vs. BG (Mann–Whitney U, *p*-value > 0.05), analysis of the overlapping DEGs between these comparisons showed that the upregulated level (log2FC) of DEGs in both the CG vs. AG _overlap_ and CG vs. BG _overlap_ exhibited significant differences (Mann–Whitney U, *p*-value < 0.01) (Figure 3c). This indicates a greater magnitude of differential expression change for B subgenome genes.

Analysis of subgenome-biased differential expression among AG, BG, and CG revealed that 31.83% of genes maintained balanced expression in AG vs. BG, while this proportion increased to 40.62% in BG vs. CG (Figure 3d). This suggests that, as the severity of GRS infection increases, more genes contribute to the restoration of transcriptional homeostasis. Concurrently, CG exhibited a significant shift in subgenome dominance, with a gain in B subgenome bias being the primary expression pattern change during lesion formation. The transition from AG_balance_→CG_B-bias_ was 16.60% (1003/6043) in the DEGs of CG vs. AG, and from BG_balance_→CG_B-bias_ was 16.05% (957/5962) in the DEGs of CG vs. BG. In contrast, the frequencies for AG_balance_→CG_A-bias_ and BG_balance_→CG_A-bias_ were 11.30% (683/6043) and 11.36% (677/5962), respectively. This biased shift resulted in DEGs with B-bias accounting for 26.01% (CG vs. AG) and 25.33% (CG vs. BG) of total DEGs, which is higher than DEGs with A-bias (22.37% and 22.09%) (Figure 3d and Figure S4). This indicates BG showed characteristics of early transcriptional reprogramming compared to AG, with the proportion of DEGs with B-bias increasing to 26.4% and DEG with balance decreasing to 31.80%, suggesting preferential expression of the B subgenome at the initial stage of disease progression (Figure 3d and Figure S4).

Dynamic transcriptional bias shifts correlated with infection severity. GO enrichment analysis of genes transitioning from a balanced expression pattern to B-bias, or from A-bias to B-bias, revealed that B-biased genes were primarily enriched in transmembrane receptor signaling pathways, including transmembrane receptor serine/threonine kinase activity and cell surface receptor signaling pathways (Figure 3e). These pathways are closely related to stress responses, where extracellularly localized receptor kinases (RLKs) and downstream kinase cascades (MAPK) are likely involved in pathogen defense and abiotic stress adaptation. Remarkably, the transition from A-bias to B-bias in BG vs. CG suggests that infected phloem tissues enhance stress signaling through systemic transcriptional reprogramming to inhibit further infection progression. In summary, these results demonstrate a synergistic amplification of B-biased differential gene expression from BG to CG, positioning the B subgenome as a primary driver of GRS-induced transcriptional reprogramming and a critical functional determinant in plant–pathogen interface formation.

### 2.4. Dissecting the Mechanism of Splicing Plasticity-Regulating Multidimensional Transcriptional Networks in Ginseng Phloem

**Figure 4:**
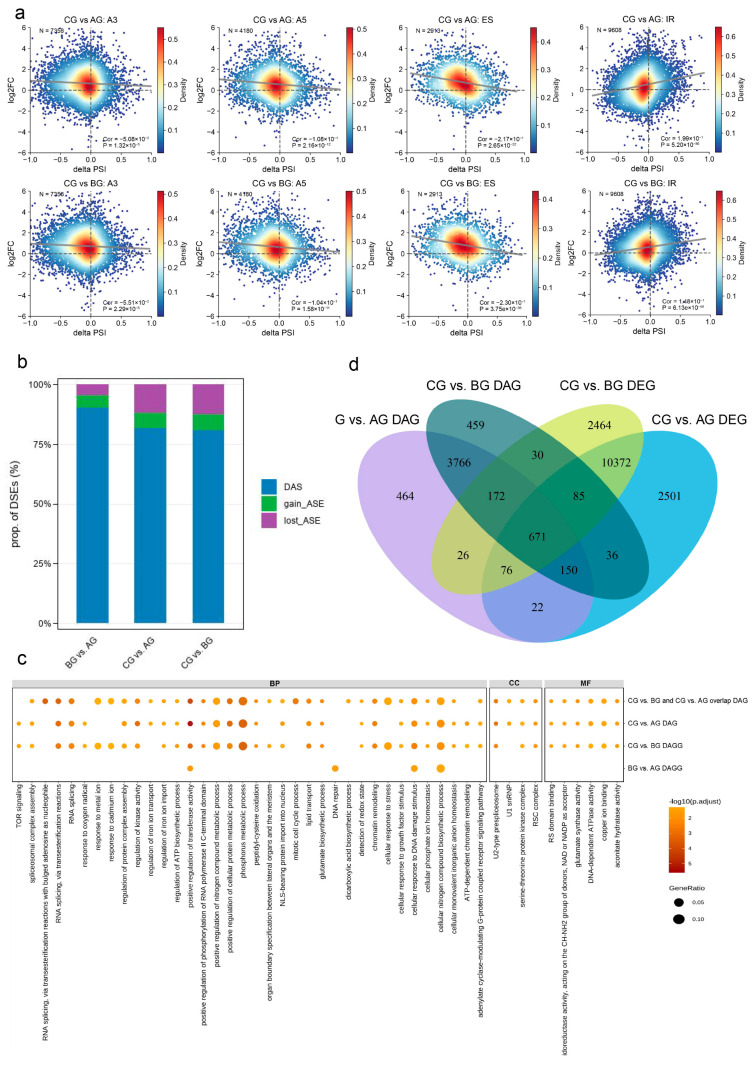
Integrated landscape of alternative splicing (AS) dynamics and gene expression changes in ginseng roots challenged by GRS. (**a**) Correlation between AS events and gene expression variation in AG, BG, and CG. Scatter-density plots show the relationship between the change in splice use (ΔPSI, *x*-axis) and the corresponding change in gene expression (log2FoldChange, *y*-axis) for alternative acceptor (A3), alternative donor (A5), exon skipping (ES), and intron retention (IR). Each point represents a single AS event and is colored by point density; the black line is the linear regression, and grey dashed lines mark ΔPSI = 0 and log2FoldChange = 0. The upper-right corner of each panel lists the number of events (n), Spearman’s correlation coefficient, and the associated *p*-value. (**b**) Quantitative–qualitative composition of differentially spliced events (DSEs). Stacked bars summarize the proportions of three DSE categories: DAS (blue), events showing only a quantitative change in splice ratio (PSI); gain_ASE (green), splice isoforms newly detected in the comparison; and lost_ASE (purple), splice isoforms absent in the comparison. Bar height corresponds to the percentage of each category within the total DSEs. Events were designated as DSEs when |ΔPSI| ≥ 10% with *p*-value < 0.05. (**c**) Functional enrichment of AS-regulated genes in AG, BG, and CG. Bubble plots illustrate Gene Ontology terms significantly enriched among AS genes. Bubble size denotes the GeneRatio, whereas color intensity represents –log10 (adjusted *p*-value). (**d**) Overlap between differentially expressed genes (DEGs) and differentially spliced genes (DAGs). The Venn diagram compares DEGs and DAGs identified in CG vs. AG and CG vs. BG. Circles correspond to CG vs. AG DAGs (purple), CG vs. BG DAGs (dark green), CG vs. BG DEGs (yellow), and CG vs. AG DEGs (blue), and numbers indicate gene counts for each subset.

Correlation analysis between the ΔPSI of four differential alternative splicing events (A3, A5, ES, and IR) and gene expression in CG vs. AG and CG vs. BG contrasts revealed that most of the transcripts clustered around the origin of the two-dimensional density plot, indicating broadly stable splicing patterns and expression levels. However, significant deviations were observed at the edges of the plot, suggesting a subset of genes undergoing coordinated shifts in both parameters (Figure 4a). In the CG vs. AG comparison, the ΔPSI values for A3, A5, and ES events showed weak to moderate negative correlations with gene expression changes (r = −0.051, −0.108, and −0.217, respectively; *p*-value < 0.001). This suggests that, for these AS events, an increase in the relative abundance of the alternative isoform (indicated by a positive ΔPSI) is associated with the transcriptional downregulation of the host gene (Figure 4a). IR showed the opposite trend (*r* = 0.199; *p*-value < 0.001), suggesting that increased intron retention in CG is associated with upregulated expression, potentially promoting the transcript output or translation of a class of stress/defense-related genes (Figure 4a). The same analysis for CG vs BG showed lower correlation coefficients (*r*_A3_ = −0.056, *r*_A5_ = −0.104, *r*_ES_ = −0.230, *r*_IR_ = 0.148), consistent with the notion that lesion-adjacent tissue has already initiated a degree of splice expression coregulation prior to developing into the necrotic core (Figure 4a).

We then simultaneously performed quantitative (ΔPSI) and qualitative (de novo gain and loss of DSEs) analyses of differential splicing events (DSEs). In the BG vs. AG comparison, the frequency of DAS (differentially alternatively spliced) events was 90.57%, while the gain-ASE and lost-ASE frequencies were 4.9% and 4.53%, respectively. This indicates that splicing differences in BG relative to AG primarily involved conventional exon/intron boundary selection. In both the CG vs. AG and CG vs. BG contrasts, however, the proportion of DAS events declined, whereas the proportions of gain-ASE and lost-ASE events increased (Figure 4b), indicating extensive remodeling of the splicing landscape in the core lesion, where previously latent isoforms are silenced, whereas novel splice forms emerge. Notably, the gain/lost-ASE proportions in CG vs BG closely resembled those in CG vs AG, implying that these splicing alterations are specific to the lesion core rather than gradual changes that transition with tissue progression. This indicates a hierarchical response in splicing regulation, with initial interventions of a quantitative level, followed by alternative remodeling in the core region with gain/lost-AS events.

The overlap between alternatively spliced genes in the CG vs AG and CG vs BG contrasts (Figure S5), suggesting a conserved post-transcriptional regulatory mode accompanying GRS lesion formation. GO enrichment analysis revealed that overlapping AS genes were significantly enriched in RNA splicing regulation (e.g., spliceosomal complex assembly and RNA trans-splicing) and stress response pathways (e.g., oxidative stress and cadmium ion detoxification). Further analysis dissecting enrichment based on AS event type revealed specific functional associations. IR events were notably enriched in pathways related to carbohydrate and nucleotide metabolism (e.g., the carbohydrate derivative biosynthetic process and nucleotide phosphorylation) and auxin polar transport, suggesting a role in metabolic adjustment and hormonal signaling. A3 events were associated with stress response pathways, including the cellular response to organonitrogen compounds and response to iron ion starvation, implying they could be involved in adaptive responses to various stresses. A5 events showed strong enrichment in nitrogen metabolism pathways (e.g., the ammonia assimilation cycle and glutamine metabolic process) (Table S6). Additionally, AS events related to chromatin remodeling (ATP-dependent chromatin remodeling) and metabolic pathways (ATP biosynthesis and phosphate metabolism) were also significantly enriched, as well as the TOR signaling pathway and serine/threonine kinase activity, suggesting regulatory nodes for the growth–defense balance. These results demonstrate the multidimensional regulatory role of AS to stress adaptation and cellular homeostasis (Figure 4c).

The comparison of DEGs and DAGs identified 3464 DEGs and 459 DAGs in the CG vs. AG comparison, with 172 genes regulated by both layers. In the CG vs BG comparison, 3010 DEGs and 85 DAGs overlapped in 26 genes, and 671 overlapped genes were identified among all four datasets (Figure 4d). Functional enrichment analysis of 671 genes, representing key regulatory nodes under dual transcriptional and post-transcriptional control, revealed significant enrichment predominantly within the 606 upregulated genes. These upregulated genes were highly enriched in catabolic processes, particularly those involving macromolecule degradation, such as the modification-dependent macromolecule catabolic process, ubiquitin-dependent protein catabolic process, and proteolysis involved in cellular protein catabolic process. Additional enriched pathways included the response to endoplasmic reticulum stress, RNA repair, and regulation of protein ubiquitination. Conversely, the 65 downregulated coregulated genes did not yield any significant GO term enrichment. These findings suggest that the coordinated transcriptional upregulation and AS of these genes may drive the stress management within the lesion core (Table S7). Functional annotation reveals roles in cell-cycle control (CDC5 and RPN1A), transcription and splicing (NF-YB8 and U2AF65b), DNA repair and chromatin remodeling (REV3 and MORC6), stress adaptation (ANNAT8 and HCC2), hormone signaling (DCAF1), cell-wall modification (PAE3), photosynthesis (PHT4;3), and pathogen interaction (MIM). Their concerted transcriptional and splicing regulation suggests that they coordinate growth, development, and defense as GRS lesions mature (Table S8).

## 3. Discussion

This study provides the first genome-wide characterization of alternative splicing (AS) dynamics in *P. ginseng* roots during rusty root disease (GRS), complementing earlier metabolomic and hormonal work that largely overlooked post-transcriptional regulation [9,36]. We observed that heavily infected tissues (CG) display a marked increase in both AS events and affected genes, underscoring the widespread spliceosome activation under pathogen pressure. In plants, AS is now recognized as an integral component of immunity and stress responses [27,29,31,37]. The predominance of intron retention (IR) we observe mirrors many stress-responsive datasets [34,38,39] and aligns with the model in which IR transcripts are retained in the nucleus and released for translation upon rising defense signals [40]. *Arabidopsis* immune receptors such as *RPS4* and *RPP4* exemplify this mechanism, producing IR isoforms that bypass nonsense-mediated decay and can be rapidly exported upon infection [41,42]. By contrast, exon skipping (ES) and alternative splice-site choices (A3/A5) are frequently associated with transcript downregulation or truncation [43], acting as a molecular brake on energetically costly genes and, in some pathosystems, being hijacked by pathogens to weaken host defenses [17]. It is worth noting that our AS identification relies on short-read data, which can make precise isoform-level analysis challenging. While this may lead to some overestimation from low-abundance transcripts, our study provides a genome-wide overview of splicing dynamics during GRS. Nevertheless, because the present analysis is based exclusively on Illumina-derived short reads and lacks orthogonal experimental confirmation (e.g., RT-PCR, Sanger validation, or functional assays), the continuity of individual isoforms and the accuracy of specific splice junctions remain provisional.

Pathogens can also directly affect host AS. The Phytophthora infestans effector IPI-O1 (Avrblb1) binds the potato spliceosomal protein StCWC15, promoting productive splicing of the resistance gene *RB* and enhancing late-blight immunity [29]. Similar splicing network reorganization has been reported for the *SR45a* splicing factor during *Verticillium* infection of cotton, where *SR45a* self-splicing modulates the processing of multiple immune genes [30]. Collectively, these observations underscore the dual nature of AS, as a defense amplifier and a pathogen target, whose net effect depends on the molecular arms race in each host–microbe interaction [17,37].

*P. ginseng* is an ancient polyploid in which the A and B subgenomes have undergone divergent evolutionary paths. This divergence raises the question of whether the two subgenomes use distinct defense mechanisms against rusty root disease, potentially explaining the subgenome bias observed in our study. Subgenome bias is a recurring theme in polyploid adaptation, for example, in wheat immunity and cotton [44]. Under severe GRS, the B subgenome contributes disproportionately to defense-related transcripts, indicating functional partitioning of homoeologous genes. Among the overlapping AS differentially expressed genes we identify, ranging from the cell-cycle regulator CDC5 to the stress sensor ANNAT8 and chromatin remodeler MORC6, both AS and subgenome bias cooperatively sculpt a multilayered defense program that balances growth with immunity. Targeting splice-regulatory nodes within the dominant B subgenome therefore offers a promising route to enhance GRS resistance.

Our chromosomal heatmaps revealed AS hotspots in gene-rich distal arms (e.g., Chr04A, Chr08A, and Chr10B), suggesting that local genomic architecture modulates splice-site choice. Accumulating evidence links chromatin features, exon–intron GC differences, and chromatin loops with splice-site recognition in plants [45]. We therefore propose that the AS hotspots documented here reflect underlying chromatin states that facilitate recurrent recruitment of spliceosomes, a hypothesis testable by profiling histone marks and chromosome conformation capture in future studies.

Future research should leverage on third-generation long-read sequencing platforms, such as PacBio Iso-Seq and Oxford Nanopore, to generate full-length transcriptomes, achieve precise isoform phasing, and resolve complex splicing events that remain inaccessible to short-read assemblies. These datasets require rigorous validation by targeted RT-PCR/qRT-PCR and, where feasible, functional assays (e.g., isoform-specific over-expression or CRISPR-mediated splice-site editing) to ascertain the biological relevance of the AS events identified herein and to prioritize candidate genes for breeding programs. Adoption of this integrated, multi-modal strategy will directly surmount the methodological limitations of the present study and establish a more robust framework for dissecting AS-mediated disease resistance in ginseng.

In summary, our integrated analysis uncovers a concerted interplay between AS plasticity and subgenome bias that underpins the ginseng response to GRS. These insights not only deepen our understanding of plant defense multilayers but also identify potentially valuable targets, splice-regulatory elements, and dominant-subgenome genes for breeding and biotechnological strategies aimed at durable disease resistance.

## 4. Materials and Methods

### 4.1. Transcriptome Data Acquisition

This study utilized publicly available RNA sequencing data of ginseng root rusty symptoms (GRSs), encompassing healthy control (AG) and ginseng root phloem tissues with varying degrees of GRS infection (BG: lesion-adjacent and CG: lesion-core) [36]. All raw RNA-Seq data (FASTQ format) were retrieved from the National Center for Biotechnology Information Sequence Read Archive (NCBI SRA) under BioProject ID: PRJNA993718. The sequencing was performed on an Illumina NovaSeq 6000 high-throughput sequencer (Illumina, San Diego, CA, USA). The published *P. ginseng* T2T genome sequence (BioProject ID: PRJCA022032) was used as the reference genome [5].

### 4.2. Data Preprocessing

Raw sequencing reads underwent quality control using the Trimmomatic software (v0.39) [46]. Bases with quality scores below Q10 were filtered, and adapter sequences were removed to obtain high-quality data for subsequent alignment. Subsequently, the high-quality reads were mapped to the *P. ginseng* T2T reference [5] genome using the STAR (v2.7.11b) [47] aligner with the following parameters: --runThreadN 20 --genomeDir --readFilesCommand zcat --readFilesIn $fit1 $fit2 --outFileNamePrefix $star_out”/”$pref --limitBAMsortRAM 100000000000 --outSAMstrandField intronMotif --outSAMtype BAM SortedByCoordinate --quantMode GeneCounts --outReadsUnmapped Fastx --chimSegmentMin 15 --chimJunctionOverhangMin 15 --twopassMode Basic --outSAMattrIHstart 0 --outSAMattributes NH HI AS nM NM MD XS --alignEndsType EndToEnd. SAMtools was then employed to filter for reads with high mapping quality (Map Quality > 20) for downstream analyses [48].

### 4.3. Transcript Assembly and Annotation

Transcript assembly was performed for each sample’s three biological replicates using the Scallop assembly software (v0.10.4) [49] with the parameters --min_transcript_coverage 3 and --min_flank_length 5. Based on the splice junction information generated by the STAR alignment, a custom Python (v3.8.19) script was used to filter out low-abundance transcripts with fewer than three supporting reads in each replicate. Subsequently, the StringTie software (v2.1.7) [50] was used to merge the assembled transcripts from each replicate (parameters: --merge -i, -f 0.05, -T 1, -F 1, -m 200). Gffcompare (v0.11.2) [51] was then utilized to compare the merged transcripts with the reference genome annotation, extracting splice pattern annotations. Only transcripts with class codes “=“ or “j” were retained for downstream analysis, resulting in a total of 92,082 transcript isoforms.

### 4.4. Identification of Gene Alternative Splicing Events

The SUPPA2 software (v2.4) [52] was used to identify gene alternative splicing events based on the merged annotation file. Four major types of AS events were considered: intron retention (IR), exon skipping (ES), alternative donor (A5), and alternative acceptor (A3). To minimize false positives, a custom Python script was applied to filter initial AS events, integrating data from all sample replicates and discarding any event supported by ≤3 reads in every replicate of at least one experimental group.

### 4.5. Differential Splicing Event Analysis

The Percentage Spliced In (PSI) was calculated for each AS event in each sample to quantify splicing patterns and compare inter-group differences. PSIs range from 0 to 1, representing the proportion of a specific exon being spliced into the mature transcript. Initially, the number of AS events with 0 < PSI < 1 in each sample was tallied to ascertain the overall changes in AS events in healthy and infected tissues. Subsequently, ΔPSI (ΔPSI = PSI_CG_ − PSI_AG_) was used to quantify the splicing differences between two sample groups, and a binomial distribution test was performed based on the counts of inclusion and exclusion reads. Significant differential splicing events (DSEs) were identified using a threshold of |ΔPSI| > 0.20 and *p*-value < 0.05. The specific criteria for classification were as follows: if the control group’s PSI was nearly 0 or 1 (PSI_AG_ > 0.95 or <0.05), while the treatment group’s PSI was between 0.05 and 0.95, the AS event was considered “gained”; conversely, if the infected group’s PSI was close to 0 or 1 and the control group’s PSI was in the intermediate range, the event was classified as “lost”. Other events satisfying the threshold but not representing complete gain or loss were categorized as “effective alternative splicing events (DAS)”. This systematic approach allowed for the identification of AS alteration profiles across healthy and infected two ginseng root tissues.

### 4.6. Gene Expression and Subgenome Bias Analysis

Protein sequences encoded by *P. ginseng* transcripts were aligned against the *Arabidopsis thaliana* reference protein database using BLASTP (E-value threshold 1 × 10^−10^) to identify homoeologous annotations for ginseng genes. Differential gene expression analysis was performed using the DESeq2 software (v1.42.1) [53] based on gene read counts from each sample. Genes with |log2 (FoldChange)| ≥ 2 and an adjusted significance *q*-value < 0.05 were considered significantly differentially expressed genes (DEGs). To analyze differential subgenome responses under GRS, we extracted protein sequences for the *P. ginseng* A and B subgenomes separately using Gffread. A homoeologous gene pairing database between subgenomes was then established using makeblastdb and BLASTP. Homoeologous gene pairs with sequence similarity ≥ 90% were selected, and a custom Perl script was used to identify unique homoeologous gene pairs corresponding between the A and B subgenomes. Subsequently, a custom Python script was used to compare the expression levels of each homoeologous gene pair under different treatments, calculating the subgenome-biased expression ratio. The proportion of genes exhibiting significant bias towards either the A/B subgenome in the GRS-infected group was then statistically analyzed to assess the role of subgenome bias (“B-bias”) in the GRS response.

### 4.7. Gene Ontology Enrichment Analyses

The genome annotation file was downloaded from the article “Telomere-to-Telomere Reference Genome for *Panax ginseng* Highlights the Evolution of Saponin Biosynthesis” [5]. GO enrichment analyses were performed using the R program with hypergeometric distribution tests, and an FDR < 0.05 was used as the significance threshold for differential enrichment. The GeneRatio (N/M) represents the ratio of the number of genes from our gene list found in a specific pathway (N) to the total number of genes annotated to that same pathway in the genomic background (M).

## Data Availability

Data are available in the article’s Supplementary Materials.

## Supplementary Materials

The following supplemental information can be downloaded at https://www.mdpi.com/article/10.3390/plants14142120/s1: Figure S1. Alternative splicing (AS) patterns in three *P. ginseng* samples (AG, BG, and CG); Figure S2. Correlation heatmaps of PSIs for four alternative splicing event types (IR, A3, A5, and ES) in AG, BG, and CG; Figure S3. Correlation analysis of alternative splicing (AS) event count and isoform count per gene in AG, BG, and CG samples; Figure S4. Dynamic analysis of subgenome gene expression bias; Figure S5. Venn diagram of differentially alternative spliced genes (DAGs) for CG vs. AG and CG vs. BG; Table S1. The number of AS events in three samples; Table S2. The gene number of AS events in three samples; Table S3. Number of ASEs (alternative splicing events) in each ASG (alternative splicing gene); Table S4. Number of ASEs (alternative splicing events) in A/B subgenomes; Table S5. Number of ASGs (alternative splicing genes) in A/B subgenomes; Table S6. The GO terms of four types in AS events (IR, A3, A5, ES); Table S7. GO Enrichment of 671 Overlapping DEGs and DAGs between CG vs. AG and CG vs. BG; Table S8. Orthologous genes between *P. ginseng* and *Arabidopsis Thaliana*.
