# Supplementary material for: Dynamic Coordination of Alternative Splicing and Subgenome Expression Bias Underlies Rusty Root Symptom Response in Panax ginseng
Source: Plants (Basel). 2025 Jul 9;14(14):2120. doi: 10.3390/plants14142120 (PMC12298674; doi:10.3390/plants14142120)
Supplement: Supplementary file 1 [file plants-14-02120-s001.zip › Supporting Information.pdf]

Supporting Information

Article title: Dynamic coordination of alternative splicing and subgenome expression bias underlies rusty root symptom response in *Panax ginseng*

Jing Zhao[1,4‡], Juzuo Li[5‡], Xiujuan Lei[1,4], Peng Di[1,4], Hongwei Xun[6], Zhibin Zhang[6], Jian Zhang[1,2,3*], Xiangru Meng[1,4*], Ying-Ping Wang[1,4*].

The following Supporting Information is available for this article:

Figure S1. Alternative Splicing (AS) Patterns in Three *Panax ginseng* Samples (AG, BG, CG).

Figure S2. Correlation Heatmaps of PSIs for Four Alternative Splicing Event Types (IR, A3, A5, ES) in AG, BG, and CG.

Figure S3. Correlation Analysis of Alternative Splicing (AS) Event Count and Isoform Count per Gene in AG, BG, and CG Samples.

Figure S4. Dynamic Analysis of Subgenome Gene Expression Bias.

Figure S5. Venn Diagram of Differentially Alternative Spliced Genes (DAGs) for CG vs. AG and CG vs. BG.

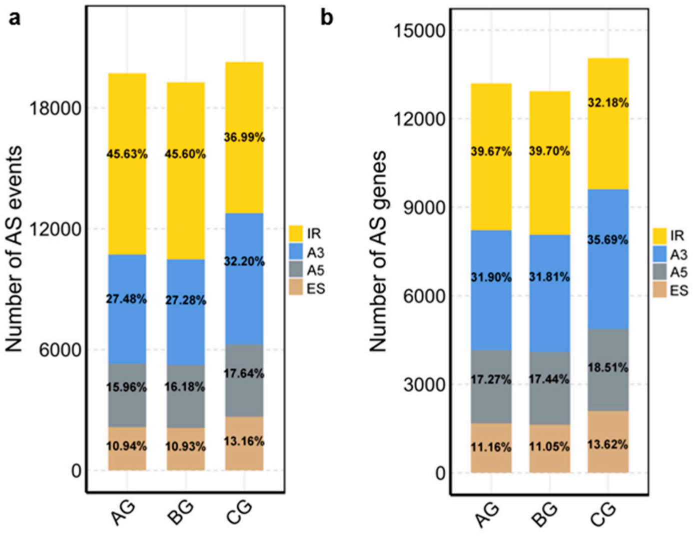

Figure S1. Alternative Splicing (AS) Patterns in Three *Panax ginseng* Samples (AG, BG, CG). (a) Number and proportion of four types of alternative splicing events: intron retention (IR), alternative acceptor (A3), alternative donor (A5), and exon skipping (ES). (b) Number and proportion of alternative spliced genes (ASGs) corresponding to these event types.

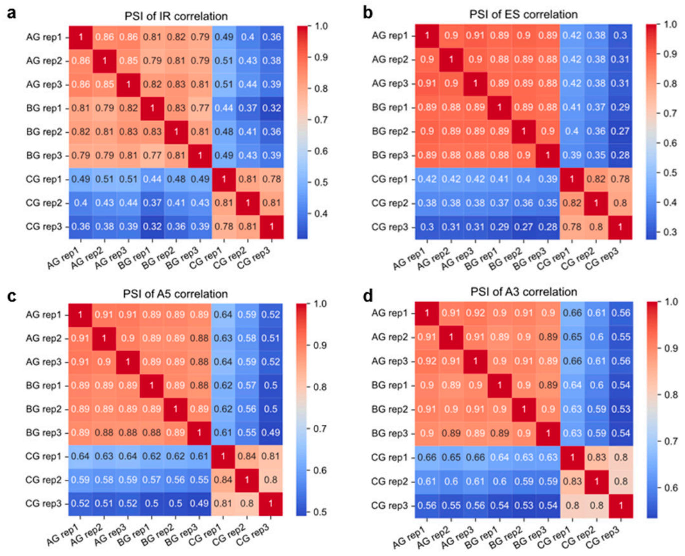

Figure S2. Correlation Heatmaps of PSIs for Four Alternative Splicing Event Types (IR, A3, A5, ES) in AG, BG, and CG. Correlation matrices for (a) Intron Retention (IR), (b) Exon Skipping (ES), (c) alternative donor (A5), and (d) alternative acceptor (A3). Both x- and y-axes represent biological replicates of each treatment. Values within each cell

indicate the Pearson correlation coefficient (ranging from 0 to 1), with color intensity ranging from blue (low correlation) to red (high correlation).

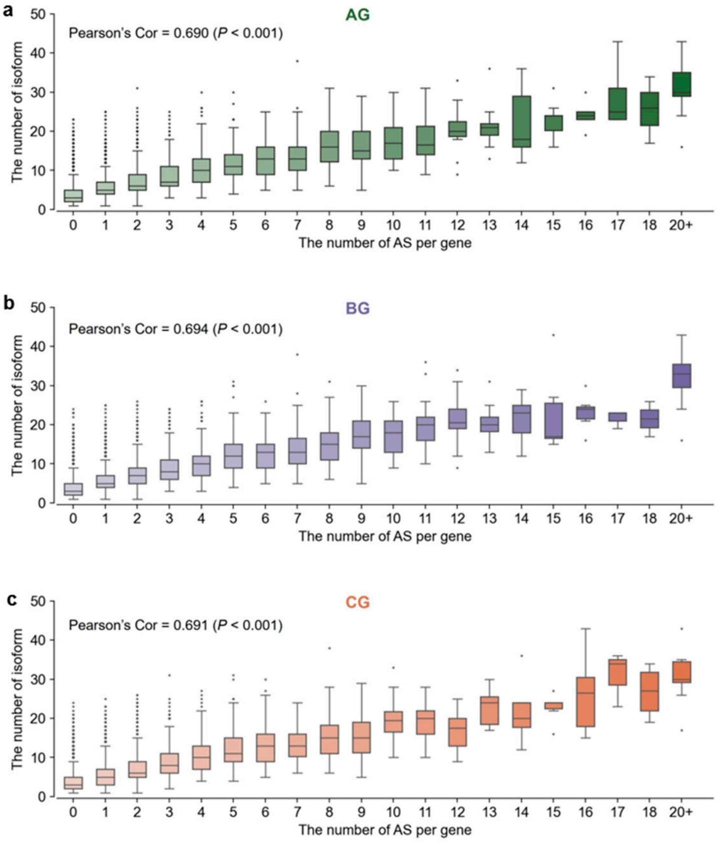

Figure S3. Correlation Analysis of Alternative Splicing (AS) Event Count and Isoform Count per Gene in AG, BG, and CG Samples. Box plots display the distribution of the number of AS events per gene (*x*-axis) and the corresponding number of isoforms (*y*-axis) across the entire genome for the three samples (AG, BG, CG). Pearson correlation coefficients and their significance levels (*p*-value) are indicated in the figure.

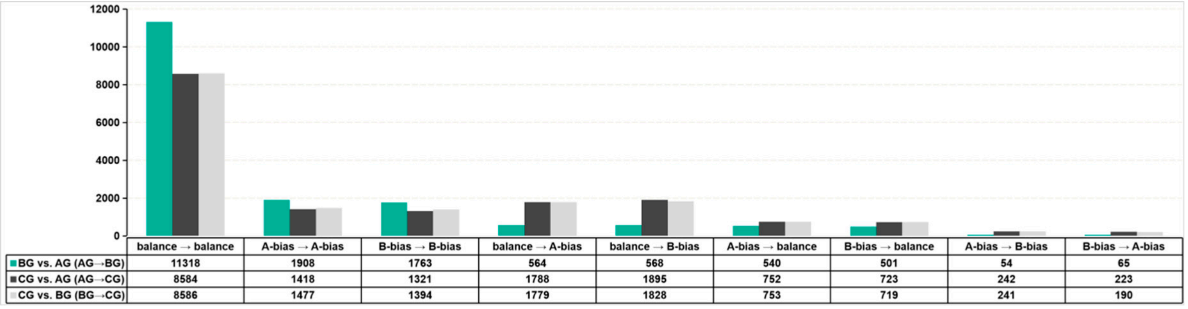

	balance → balance	A-bias → A-bias	B-bias → B-bias	balance → A-bias	balance → B-bias	A-bias → balance	B-bias → balance	A-bias → B-bias	B-bias → A-bias
BG vs. AG (AG→BG)	11318	1908	1763	564	568	540	501	54	65
CG vs. AG (AG→CG)	8584	1418	1321	1788	1895	752	723	242	223
CG vs. BG (BG→CG)	8586	1477	1394	1779	1828	753	719	241	190

Figure S4. Dynamic Analysis of Subgenome Gene Expression Bias. Transcriptome comparisons of AG, BG, and CG reveal three expression patterns: balanced (no subgenome dominance), A-biased, and B-biased. Numerical labels indicate the number of differentially expressed genes.

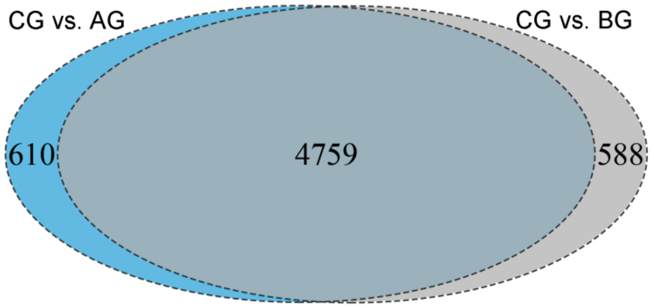

Figure S5. Venn Diagram of Differentially Alternative Spliced Genes (DAGs) for CG vs. AG and CG vs. BG. Two circle represent DAGs from CG vs. AG (blue) and CG vs. BG (grey), respectively. Numbers within the circles indicate the gene counts.
